# System introduction and evaluation of the first Chinese chest EIT device for ICU applications

**DOI:** 10.1038/s41598-021-98793-0

**Published:** 2021-09-29

**Authors:** Shuo-Yao Qu, Meng Dai, Shuo Wu, Zhi-Rang Lv, Xin-Yu Ti, Feng Fu

**Affiliations:** 1 Department of Pulmonary and Critical Care Medicine, Xijing Hospital, Fourth Military Medical University, No. 127 Changle West Road, Xi’an, 710032 China; 2 Department of Biomedical Engineering, Fourth Military Medical University, No. 169 Changle West Road, Xincheng District, Xi’an, 710032 China; 3 Northwest Machine, Xi’an, China

**Keywords:** Biological techniques, Biotechnology, Computational biology and bioinformatics

## Abstract

Chest electrical impedance tomography (EIT) is a promising application which is used to monitor the ventilation and perfusion of the lung at the bedside dynamically. The aim of the study was to introduce the first Chinese made chest EIT device for ICU application (Pulmo EIT-100). The system design of the hardware and software was briefly introduced. The performance of the system was compared to PulmoVista 500 (Dräger Medical) in healthy volunteers. The EIT system Pulmo EIT-100 consists of impedance measurement module, power supply module, PC all-in-one machine, medical cart and accessories. The performance of the system current source and voltage measurement unit was tested. A total of 50 healthy lung volunteers were prospectively examined. Subjects were asked to perform repetitive slow vital capacity (SVC) maneuvers with a spirometer. EIT measurements were performed in the following sequence during each SVC with: (1) Pulmo EIT-100, (2) PulmonVista500, (3) Pulmo EIT-100 and (4) PulmonVista500. Linearity and regional ventilation distribution of the reconstructed images from two devices were compared. The output frequency stability of the current source was 2 ppm. The amplitude error within one hour was less than 0.32‰. The output impedance of the current source was about 50kΩ. The signal-to-noise ratio of each measurement channel was ≥ 60 dB. For fixed resistance measurements, the measured values drifted about 0.08% within one hour. For human subjects, the correlations between the spirometry volume and EIT impedance from two devices were both 0.99 ± 0.01. No statistical significances were found in the parameters investigated. The repeatability (variability) of measures from the same device was comparable. Our EIT device delivers reliable data and might be used for patient measurement in a clinical setting.

## Introduction

Electrical impedance tomography (EIT) is a non-invasive, radiation-free clinical imaging technique^1^. Chest EIT is one of the most promising application of such technique, which is used to monitor the ventilation and perfusion in real-time at the bedside^2^. A total of 16 or 32 electrodes in form of electrode belt or similar is attached to the thorax surface, usually at the 4th or 5th intercostal space. Small insensible alternative currents are injected to the thorax and the corresponding surface voltages are measured. Relative impedance changes are reconstructed accordingly, representing the change of lung volume within the measurement plane.

Nearly 4 decades have passed since the emerge of the technique, only a few commercial devices are available in the market including PulmoVista 500 (Dräger Medical, Lübeck, Germany), Enlight 1800 (Timpel, São Paulo, Brazil), AirTom (Bilab Healthcare, Seoul, Korea) and LuMon (Sentec, Therwil, Switzerland). Therefrom only one is available in China. Nevertheless, the clinical research and daily usage of chest EIT in China are intensive^3^. Especially due to the outbreak of COVID-19, a Chinese guideline on COVID-19 treatment was issued and EIT was mentioned as one of the mean to titrate PEEP. A recent randomized controlled trial has demonstrated that EIT-guided titration of positive end-expiratory pressure could significantly improve the survival rate in moderate to severe acute respiratory distress syndrome compared to pressure–volume loop^4^. Hence, more and more hospitals and doctors are interested in this technique. Unfortunately, the price of the commercial device in China is too expensive for most of the hospitals to afford, especially no reimbursements of the device and measurements are possible at the moment, which prohibits the widely use of chest EIT in clinical practice.

Our group has recently developed the first Chinese chest EIT device for ICU applications. In the present study, the system design of the hardware and software was briefly introduced. The performance of the system was compared to PulmoVista 500 in healthy volunteers.

## Methods

### System architecture

**Figure 1:**
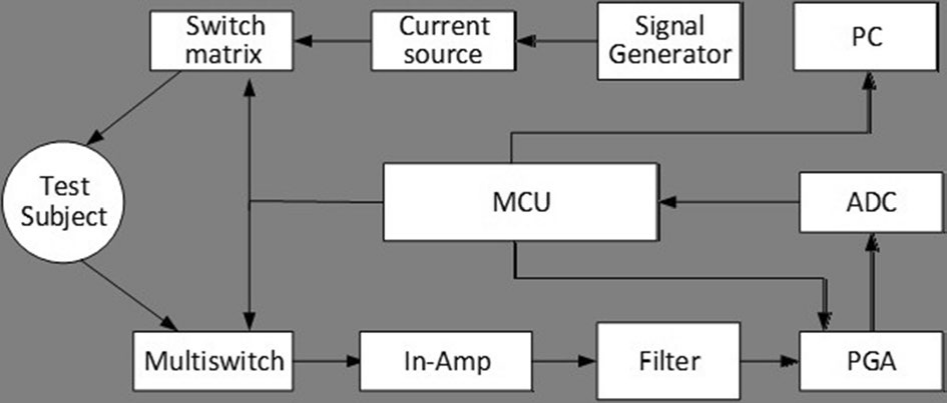
Overall design of the system. *PC* personal computer, *ADC* analog–digital converter, *PGA* programmable gain amplifier, *MCU* microcontroller unit, *In-Amp* instrumentation amplifier.

The EIT system Pulmo EIT-100 (Fourth Military Medical University, FMMU, Xi’an, China) consists of five main parts, which are impedance measurement module, power supply module, PC all-in-one machine, medical cart and accessories. The impedance measurement module is the main part of the system, including the control system unit, communication interface management unit, current source, and voltage measurement unit. The EIT system contains 16 measurement channels. The overall functions and configuration of the system are controlled through a PC. The main board contains two processors. One processor is responsible for data pre-processing and communication. The other one is for current signal generation and voltage measurement. The overall design is illustrated in Fig. 1.

### PC and software introduction

**Figure 2:**
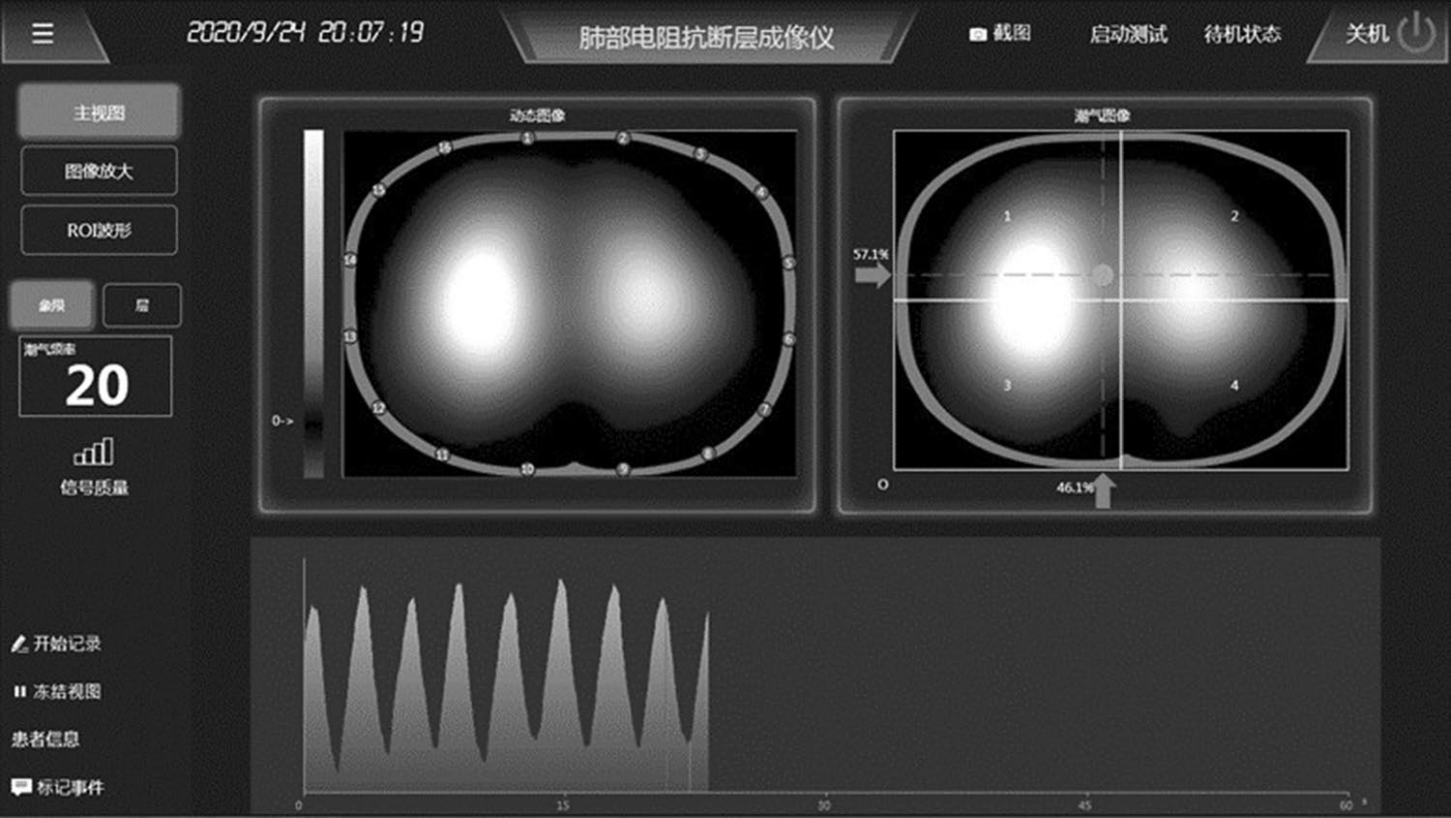
Screenshot of the software display.

The PC with Ethernet port (RJ45) is used as the imaging and control device. The PC exchanges commands and data with the lower computer through the high-speed Ethernet port. Our human–machine interface mainly displays the lung impedance time difference image, and the region of interest can be selected for sub-regional display of the impedance waveform. A screenshot of our software display is shown in Fig. 2.

### Measurement board main control unit

The function of the measurement main board (PCB) is mainly completed by MCU and FPGA control. The former is responsible for the overall scheduling of the lower computer system. It establishes communication with the PC to receive commands and configuration information from the upper computer. It also controls the FPGA for the operation of the measurement function and transmits the measurement information from the FPGA to the PC for image reconstruction.

### Current generator

The demodulation method of PulmoEIT-100 is digital. The current generation unit adopts the driving form of dual current sources, which generate 50–200 kHz single-frequency sine wave current source with a phase difference of 180°. There is a 20 MHz synchronous clock source inside the FPGA to provide synchronous clock signal to other modules. In total it has 16 channels and the frame rate can be up to 50 fps.

**Figure 3:**
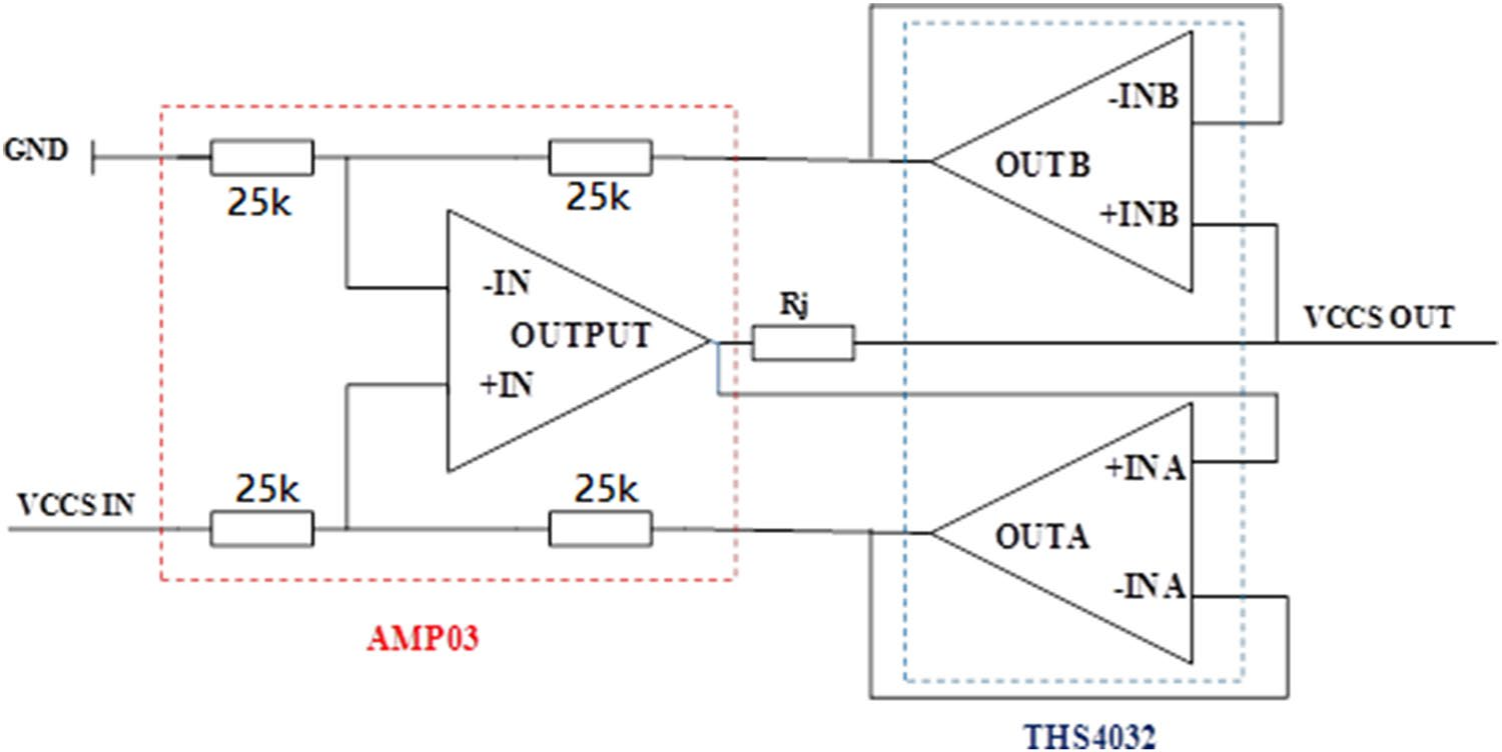
Illustration of the VCCS module circuit principle.

Th VCCS module circuit principle is illustrated in Fig. 3. The V/I converter can achieve accurate constant current output with wide bandwidth and high output impedance. The signal output from DAC is buffered and filtered into VCCS module for voltage source to current source conversion. The system adopts three op-amps VCCS circuit (the selected op-amps are AMP03 and TH4032). The working bandwidth of AMP03 is 3 MHz. Its amplification setting resistors are built-in, which can better ensure the accuracy of constant current source.

### Voltage acquisition unit

The voltage acquisition unit consists of differential amplifier, bandpass filter and gain amplifier. It converts the differential voltage between a pair of electrodes into a single-ended signal. Further, it carries out band-pass filtering to minimize DC component and high-frequency noise to ensure the accuracy and stability of the acquired signal.

### System performance evaluation

The performance of the system current source and voltage measurement unit was tested. For the current source, the frequency stability, current stability and output impedance of the output current signal were examined. For the voltage measurement unit, the signal-to-noise ratio (SNR) and stability were examined. To be specific, the device was connected to resistive network and the resistance between every pair channel was 100 Ω. The injected current was set to 5 mA and 100 kHz. Data were recorded continuously for 1 h. The SNR was calculated according to Eqs. (1) and (2):

1 $${\mathrm{SNR}}_{i}= -20\mathrm{lg}\left(\frac{\sqrt{\frac{1}{N-1}\sum_{n=1}^{N}{\left({V}_{n}-\overline{V}\right)}^{2}}}{\overline{V}}\right)$$

2 $$\overline{\mathrm{SNR}} = \frac{1}{{ch}_{max}} \sum_{i=1}^{{ch}_{max}}{SNR}_{i}$$

where i is the channel number; N is the total number of testing sample (N = 1000 in the present study); *V*_*n*_ is the voltage of the *n* testing sample; $\overline{V}$ is the average of *V*; *ch*_*max*_ is the number of total channels. The current frequency was tested with Fluke 8845A 6.5 Digit Precision Multimeter (FLUKE, Everett, US).

Further, the system performance was evaluated on human subjects. The study protocol was approved by the ethics committees of the Fourth Military Medical University (KY20213003-1) and all subjects signed the informed consent form before the experiment. A total of 50 healthy lung volunteers were prospectively examined (male:female 33:17; age, 47 ± 15 years; height, 167 ± 8 cm; weight, 66 ± 9 kg). Subjects were asked to perform repetitive slow vital capacity (SVC) maneuvers^5^ with a spirometer (HI-101; CHEST M.I., INC., Tokyo, Japan). EIT measurements were performed in the following sequence during each SVC with: (1) Pulmo EIT-100 from FMMU, (2) PulmonVista500 from Dräger Medical, (3) Pulmo EIT-100 and (4) PulmonVista500. The electrode belts from the devices were attached and detached from the subjects’ thorax, ~ 4th intercostal space. The level of the electrode plane was marked to ensure the repeatability of electrode placements for every measurement. Relax stable tidal breathing and functional residual capacity level were observed before the SVC maneuver was conducted. Sampling rate of both devices was set to 50 Hz. GREIT algorithm^6^ was used for offline analysis of EIT data from both devices to eliminate the influence of reconstruction methods.

Tidal variation images (the difference between end-inspiration and end-expiration) were calculated. Impedance values were normalized to the corresponding SVC in milliliters. The clinically widely EIT parameters were used to evaluate the differences of the measured data^7^. In brief, the following measures are calculated:

(a-b) linearity, i.e. the ratio between the tidal variation and impedance changes obtained during SVC, as well as the correlation between volume and impedance changes in individual subjects. Linear interpolation was applied for spirometry data to match the number of data points recorded by EIT.

(c-d) global ventilation distribution, indicated by the tidal variation in the right and left lungs, as well as in the ventral and dorsal regions.

(e–f) spatial ventilation distribution, indicated by the global inhomogeneity (GI) index^8,9^ and the center of ventilation (CoV)^9–11^.

(g) temporal ventilation distribution, indicated by standard deviation of regional ventilation delay (RVD)^12,13^.

### Statistical analysis

Two-sample equivalence test was conducted used to compare the differences of EIT measures between two devices. Bland–Altman plots were used to illustrate the differences. Differences between two measurements from the same devices were divided by the average to show the repeatability of the maneuver. A p value < 0.05 was considered statistically significant. EIT data and statistical analysis were performed using MATLAB R2015a (the MathWorks Inc., Natick, MA). Since no information regarding the mean and standard deviation of the EIT measures in healthy volunteers was available, sample size was predefined. The post-hoc power was calculated based on the global ventilation distribution indicated by the tidal variation in the right and left lungs.

### Ethics approval

All methods of this study were carried out in accordance with the relevant guidelines and regulations of the Fourth Military Medical University. The study was approved by the Ethics Committee of the First Affiliated Hospital of the Forth Military Medical University (No. KY20213003-1).

## Results

We have selected the frequencies of 50, 80, 100, 120 kHz for current source testing. The output frequency stability of the current source was 2 ppm. Under the selected frequency, the sine wave signal was collected and analyzed. The amplitude error within one hour was less than 0.32‰. Hence, for a short period of time, the current source output fluctuation error is negligible. The output impedance of the current source was about 50 kΩ.

A resistance network phantom was used to test the voltage measurement unit. The signal-to-noise ratio of each measurement channel was ≥ 60 dB. For fixed resistance measurements, the measured values drifted about 0.08% within one hour.

**Figure 4:**
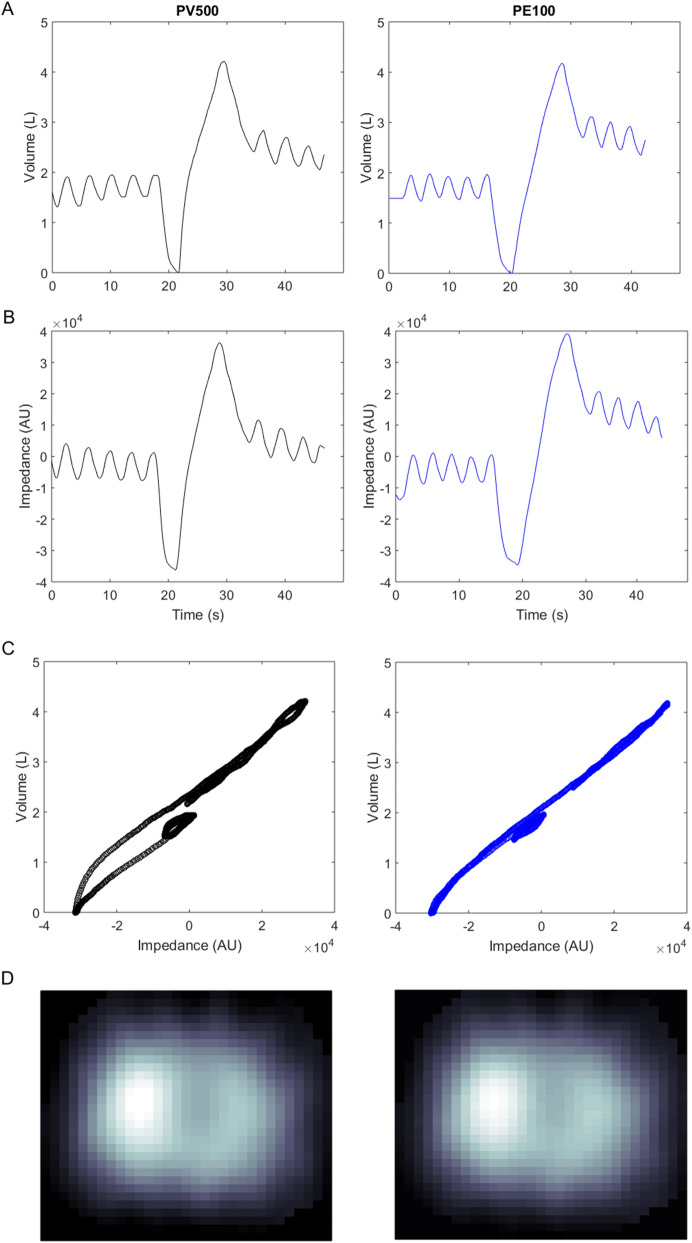
Illustration of the measurement sequence and relative impedance changes. First row, relax tidal breaths and slow vital capacity maneuvers recorded with a spirometer. Second row, impedance-time curves during the SVC maneuver. Third row, correlation between the spirometry volume and EIT impedance changes. Fourth row, impedance variation during SVC. AU, arbitrary unit. *PV500* PulmoVista 500, *PE100* PulmoEIT-100.

**Figure 5:**
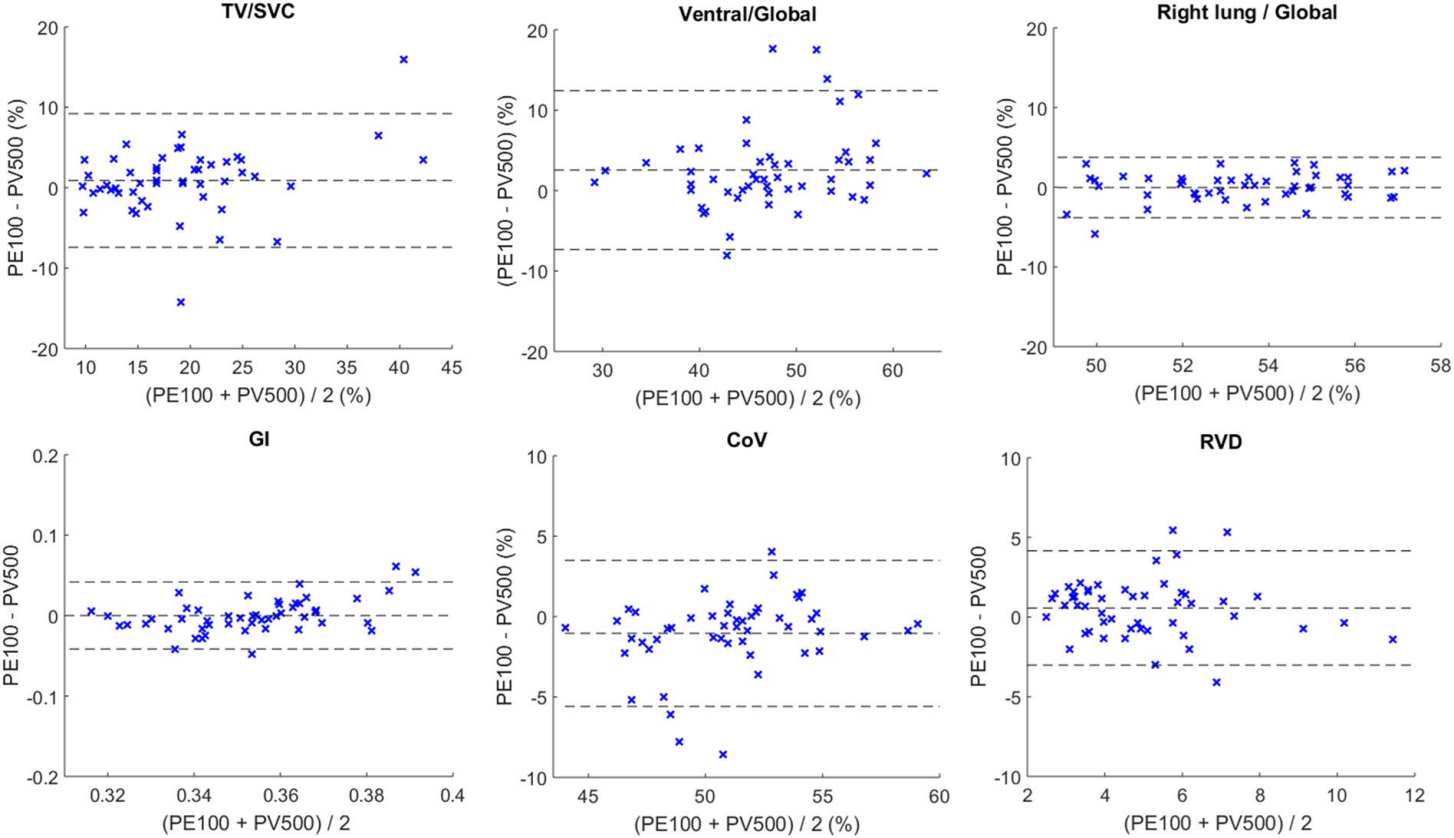
Bland–Altman plots compared the EIT-based measures calculated with PE100 (Pulmo EIT-100, FMMU) and the PV500 (PulmoVista500, Dräger). *TV* tidal impedance variation, *SVC* impedance variation during SVC maneuver, *GI* the global inhomogeneity index, *CoV* center of ventilation, *RVD* regional ventilation delay.

On human subjects, the measurements are illustrated in Fig. 4. The evaluated EIT parameters calculated based on the EIT data from PulmoEIT-100 and PulmoVista500 were similar as illustrated in Fig. 5. No statistical significances were found in the parameters. The post-hoc power calculation indicated that with sample size of 50 and α of 0.05, 1-β was 0.97. In Table 1, the repeatability (variability) of measures from the same device is summarized. No significant differences were found in the variability as well. The correlations between the spirometry volume and EIT impedance were both equaled to 0.99 ± 0.01.

**Table 1:** Variability of EIT measures from the same device.

Variability of repeated measurements with same devices	PulmoEIT100 (%)	PulmoVista500 (%)	*p*
TV/SVC	18.0 ± 12.0	17.7 ± 13.7	0.92
Volume-impedance correlation	0.6 ± 1.0	0.5 ± 0.4	0.21
Ventral to global ratio	5.6 ± 11.2	4.3 ± 4.6	0.47
Right lung to global ratio	2.0 ± 3.0	1.4 ± 1.6	0.17
GI	4.1 ± 5.4	2.8 ± 2.5	0.16
CoV	2.5 ± 6.4	1.7 ± 1.9	0.44
RVD	24.5 ± 24.3	25.1 ± 25.1	0.90

*TV* tidal impedance variation, *SVC* impedance variation during SVC maneuver, *GI* the global inhomogeneity index, *CoV* center of ventilation, *RVD* regional ventilation delay.

## Discussions

In the present study, we evaluated the first Chinese chest EIT device for ICU. The device performance is comparable to the commercial device as examined in the human subjects.

Since the ill-position problem in EIT reconstruction, the EIT devices now available are all reconstructing time-difference images. That is to say, only relative impedance can be reconstructed. Therefore, it is not possible to directly compare the impedance values of two EIT devices. In a previous study, Zhao et al. compared the EIT image analysis with different reconstruction methods^7^. In their study, instead of comparing absolute impedance values, they compared the linearity of the impedance-time curves and ventilation distribution in the reconstructed images. Similarly, in the present study, we adopted these analyses to reflect the linearity, ventilation distribution including spatial and temporal, repeatability, as well as the reliability associating with lung volume changes. The subjects haven been instructed to perform repeatable SVC maneuver to meet the ATS/ERS guideline^14^. So the EIT-based parameters are relatively repeatable (Table 1). However, the tidal breathing before the SVC was hard to control. Hence the variability of TV/SVC was higher than other parameters, except for RVD. The evaluation of RVD was conducted under low-flow maneuver^12^. Although the inspiration time during SVC is longer than normal tidal breathing, yet the length is still shorter than that during low-flow maneuver. Given the fact that the recruited subjects are all lung-healthy volunteers, the average values and the variations obtained in the study for RVD are not representing the tidal recruitment, as suggested in the clinical studies (e.g.^15,16^).

A recent paper has summarized the EIT circuits and systems available in the labs for various applications^17^. Based on the data collected in this review paper, our device has a relatively high signal-to-noise ratio and frame rate. Wu et al. has introduced a system for neonatal ventilation monitoring, which has a higher frame rate up to 122 fps^18^. The system from Draeger also has a frame rate of 50 fps, which shows no limitation in its ventilation and perfusion monitoring^19^. Besides, most of the papers that introduce novel EIT systems mainly focus on system architecture and phantom experiments. Few studies have evaluated the performance of the systems in comparison with the commercial devices. Since the final aim of the device development is the real-time applications in clinical environment. We consider that the performance of the device on human subjects should be the most important end-point of the paper.

As a limitation of this study, the long term device stability was not examined on the human subjects. Nevertheless, the hardware test of current source and voltage measurements showed that the Pulmo EIT-100 from FMMU is stable and reliable for measurements over one hour. The experiment was carried out in only one body position. Nevertheless, the comparison of our system with a widely used commercial product, PulmoVista 500 indicated that the performance of our system was comparable to PulmoVista 500 in respect of image analysis and interpretation. The performance of the proposed system should be further validated in clinical environmental settings.

## Conclusion

The EIT device from FMMU delivers reliable data and might be used for patient measurement in a clinical setting.

## Data Availability

The datasets used and/or analyzed in the present study are available from the corresponding author upon reasonable request.
